# Diversity of Rickettsiales in *Rhipicephalus microplus* Ticks Collected in Domestic Ruminants in Guizhou Province, China

**DOI:** 10.3390/pathogens11101108

**Published:** 2022-09-27

**Authors:** Miao Lu, Chao Meng, Xiang Gao, Yue Sun, Jun Zhang, Guangpeng Tang, Yilin Li, Mengyao Li, Guangyi Zhou, Wen Wang, Kun Li

**Affiliations:** 1 National Institute for Communicable Disease Control and Prevention, Chinese Center for Disease Control and Prevention, Changping District, Beijing 102206, China; 2 College of Life Sciences, Shandong First Medical University & Shandong Academy of Medical Sciences, Taian 271016, China; 3 Tongzhou Center for Disease Control and Prevention, Tongzhou District, Beijing 101100, China; 4 Guizhou Center for Disease Control and Prevention, Guiyang 550004, China; 5 Liuzhi Center for Disease Control and Prevention, Liupanshui 553400, China; 6 Tianjin Key Laboratory of Food and Biotechnology, Tianjin University of Commerce, Beichen District, Tianjin 300134, China

**Keywords:** Guizhou Province, *Rhipicephalus microplus*, *Rickettsia*, *Ehrlichia*, *Anaplasma*

## Abstract

*Rhipicephalus microplus* ticks are vectors for multiple pathogens infecting animals and humans. Although the medical importance of *R. microplus* has been well-recognized and studied in most areas of China, the occurrence of tick-borne Rickettsiales has seldom been investigated in Guizhou Province, Southwest China. In this study, we collected 276 *R. microplus* ticks from cattle (209 ticks) and goats (67 ticks) in three locations of Guizhou Province. The *Rickettsia*, *Anaplasma*, and *Ehrlichia* were detected by targeting the 16S rRNA gene and were further characterized by amplifying the key genes. One *Rickettsia* (*Ca.* Rickettsia jingxinensis), three *Ehrlichia* (*E. canis*, *E. minasensis*, *Ehrlichia* sp.), and four *Anaplasma* (*A. capra*, *A. ovis*, *A. marginale*, Ca. Anaplasma boleense) species were detected, and their *gltA* and *groEL* genes were recovered. *Candidatus* Rickettsia jingxinensis, a spotted fever group of *Rickettsia*, was detected in a high proportion of the tested ticks (88.89%, 100%, and 100% in ticks from the three locations, respectively), suggesting the possibility that animals may be exposed to this type of *Rickettsia*. All the 16S, *gltA*, *groEL*, and *ompA* sequences of these strains are 100% identical to strains reported in Ngawa, Sichuan Province. *E. minasensis*, *A. marginale*, and *Candidatus* Anaplasma boleense are known to infect livestock such as cattle. The potential effects on local husbandry should be considered. Notably, *E. canis*, *A. ovis*, and *A. capra* have been reported to infect humans. The relatively high positive rates in Qianxinan (20.99%, 9.88%, and 4.94%, respectively) may indicate the potential risk to local populations. Furthermore, the genetic analysis indicated that the *E. minasensis* strains in this study may represent a variant or recombinant. Our results indicated the extensive diversity of Rickettsiales in *R. microplus* ticks from Guizhou Province. The possible occurrence of rickettsiosis, ehrlichiosis, and anaplasmosis in humans and domestic animals in this area should be further considered and investigated.

## 1. Introduction

Ticks (Acari: Ixodida) are a group of hematophagous ectoparasites that feed on terrestrial vertebrates. They are globally distributed and divided into three major families, namely the Ixodidae, Argasidae, and Nuttalliellidae. Ticks are believed to be second only to mosquitoes as vectors for human pathogens and are the primary vectors for pathogens of wild and domestic animals [1]. They play an important role in the ecology of numerous human and animal pathogens [2,3,4,5]. Annually, tick-borne pathogens are responsible for over 100,000 cases of illness in humans worldwide [5]. Tick-borne zoonotic pathogens infecting humans include tick-borne encephalitis virus (TBEV), severe fever with thrombocytopenia syndrome virus (SFTSV), Crimean–Congo hemorrhagic fever virus (CCHFV), *Rickettsia rickettsia* (the agent of Rocky Mountain spotted fever), *R. sibirica* (the agent of Siberian tick typhus), *Anaplasma phagocytophilum* (the agent of human granulocytic anaplasmosis), *A. capra*, *Borrelia burgdorferi* sensu lato (the agent of Lyme disease), *B. persica* (the agent of relapsing fever), and *Babesia microti* (the agent of human babesiosis) [2,3,4].

*Rhipicephalus microplus* is distributed in Asia, Latin America, the Middle East, and East and South Africa [6]. It is considered to be the most important tick infesting various domestic and wild animals, and results in huge economic losses throughout tropical and subtropical regions. The preferred hosts of *R. microplus* include cattle, deer, sheep, goats, horses, and buffalo [7]. Occasionally, it also bites humans [7]. As an important vector of pathogens, *R. microplus* harbors a large variety of animal (including human) pathogens such as spotted fever group *Rickettsia* (SFGR, agents of human spotted fever), *Anaplasma phagocytophilum*, *A. capra*, *A. marginale* (the agent of bovine anaplasmosis), *Ehrlichia chaffeensis*, *E. canis* (the agent of canine ehrlichiosis), *E. minasensis* (the agent of bovine ehrlichiosis), *Babesia bigemina*, *B. bovis* (the agent of bovine babesiosis), *Borrelia* spp. (agents of Lyme disease and relapsing fever), and severe fever with thrombocytopenia syndrome virus (SFTSV) [8,9,10,11], thus resulting in infections in both humans and domestic animals.

As one of the most prevalent tick species in China, *R. microplus* has been recorded in at least 188 counties. Furthermore, it was predicted to be distributed in 678 counties of China and to potentially affect 350 million people [11]. Extensive studies have been performed on *R. microplus*-vectored pathogens in China. From 1950 to 2018, multiple human pathogens including viruses (SFTSV, Jingmen tick virus, Tacheng tick virus, and Bocavirus), bacteria (SFGR, *E. chaffeensis*, *A. phagocytophilum*, and *A. capra*), and protozoans (*B. bigemina* and *B. microti*) have been detected in *R. microplus* from China [11]. Although *R. microplus* only occasionally infests humans, it may play an important role in the natural circulation of these pathogens, thus increasing the exposure to humans and domestic animals.

*Rickettsia*, *Anaplasma*, and *Ehrlichia*, belonging to the order Rickettsiales, are important vector-borne pathogens that are mainly vectored by ticks. They infect a wide variety of mammals, including humans, and are of great veterinary and medical importance. Although there have been many studies on the molecular detection of these tick-borne pathogens in China, reports on these pathogens are remarkably scarce in Guizhou Province [12,13,14], a subtropical mountainous province with an area of 176,167 km^2^ in southwest China. To investigate the geographic distribution and species composition of *Rickettsia*, *Anaplasma*, and *Ehrlichia* pathogens in *R. microplus* ticks from this area, we collected ticks from three locations in Guizhou Province and detected these pathogens in them.

## 2. Results

### 2.1. Tick Samples

**Figure 1:**
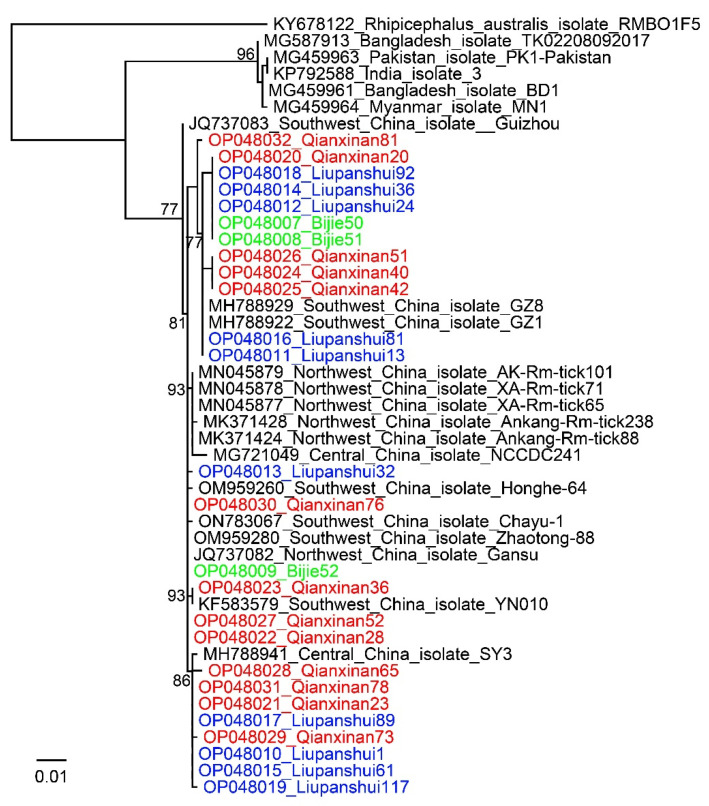
Phylogenetic trees constructed by PhyML 3.0 software (GTR model) (Sourced by Stéphane Guindon in Montpellier, France) based on the *COI* sequences (633 bp) of *Rhipicephalus microplus* ticks from three locations in Guizhou Province (3 ticks from Bijie, 10 ticks from Liupanshui, and 13 ticks from Qianxinan). Green, Bijie City; Blue, Liupanshui City; Red, Qianxinan Prefecture.

All 276 ticks were morphologically identified to be *R. microplus* by observing the capitula, legs, anal groove, and caudal appendage. All 276 ticks were fully or partially engorged. All the obtained *COI* sequences had >99% identity to *R. microplus*, confirming the morphological identification of these ticks. Phylogenetic analysis of the *COI* genes showed that the ticks formed various clades in the phylogenetic tree, indicating the polymorphism of this species in Guizhou Province (Figure 1).

### 2.2. Detection and Analysis of Rickettsia

**Figure 2:**
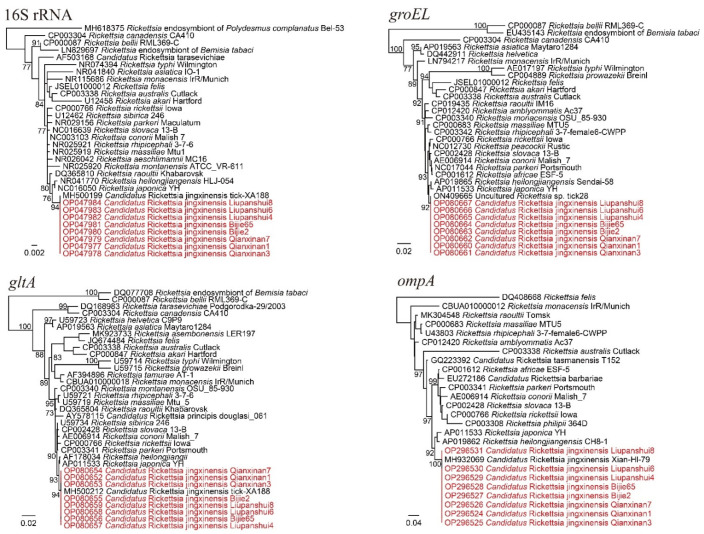
Phylogenetic trees constructed by PhyML 3.0 software (GTR model) (Sourced by Stéphane Guindon in Montpellier, France) based on the nucleotide sequences of 16S rRNA (1184 bp) and the *groEL* (1042 bp), *gltA* (985 bp), and *ompA* (706 bp) genes of *Rickettsia* strains. Red: Sequences obtained in this study.

The PCR results showed that *R. microplus* ticks from all three locations had high positive rates for *Rickettsia*. Ticks from Qianxinan, Liupanshui, and Bijie had positive rates as high as 88.89% (72/81), 100% (126/126), and 100% (69/69), respectively (Table 1). All the *Rickettsia* 16S, *gltA*, and *ompA* sequences were 100% identical to strains of *Candidatus Rickettsia jingxinensis*, a spotted fever group *Rickettsia*. For the *groEL* gene, they were 100% identical to the uncultured *Rickettsia* sp. clone tick 28 we previously identified in Ngawa in Sichuan Province, which also represents a *Candidatus* Rickettsia jingxinensis strain [15] (Figure 2). In the phylogenetic tree based on concatenated sequences (Supplementary Material Figure S1), all the strains were closely related to *Candidatus* Rickettsia jingxinensis strain. These results clearly indicated that *Candidatus* Rickettsia jingxinensis is widespread and highly prevalent in Guizhou Province.

### 2.3. Detection and Analysis of Ehrlichia

Three *Ehrlichia* species were detected and characterized, namely *E. canis*, *E. minasensis*, and *Ehrlichia* sp. *Ehrlichia canis* was detected only in Qianxinan, with a positive rate of 20.99% (17/81) (Table 1). The 16S rRNA sequences of the randomly selected strains (*E. canis* Qianxinan8 and *E. canis* Qianxinan10) had three different nucleotides and they were divided into different clades in the phylogenetic tree. However, their *gltA* and *groEL* genes were identical to each other, and they both showed the highest identity to *E. canis* strains we previously identified in Guangxi Province of Southwest China, which is adjacent to Guizhou Province [10]. *Ehrlichia minasensis* was detected in both Qianxinan and Liupanshui, with positive rates of 2.47% (2/81) and 5.56% (7/126) (Table 1). Although their 16S rRNA gene sequences had the highest (99.85–100%) similarities to the uncultured *Ehrlichia* sp. clone Honghe-42 and only 99.42–99.59% similarity to *E. minasensis* strains, the *gltA* and *groEL* sequences were both closely related to *E. minasensis* strain UFMG-EV (99.89–100% for *gltA*, 99.64–99.73% for *groEL*). In the phylogenetic tree based on concatenated sequences of these genes, all these strains were in the same clade as *E. minasensis* strain UFMG-EV (Supplementary Material Figure S1). These data support the proposal that these strains may represent an *E. minasensis* variant or recombinant.

**Figure 3:**
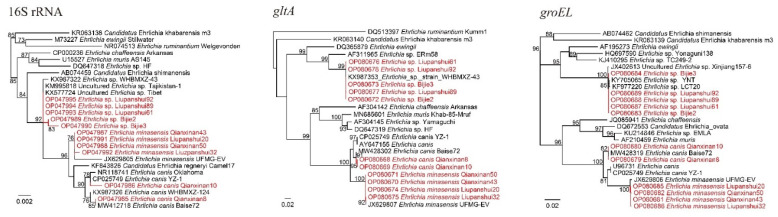
Phylogenetic trees constructed by PhyML 3.0 software (Sourced by Stéphane Guindon in Montpellier, France) (GTR model) based on the nucleotide sequences of 16S rRNA (1119–1250 bp) and the *gltA* (920–933 bp) and *groEL* (1113 bp) genes of *Ehrlichia* strains. Red: Sequences obtained in this study.

Except for *E. canis* and *E. minasensis*, an uncultured *Ehrlichia* species was detected in Bijie and Liupanshui, with positive rates of 10.14% (7/69) and 3.97% (5/126), respectively (Table 1). Genetic analysis indicated that the 16S gene sequences of the strains from Bijie have 99.76–99.92% identity to an uncultured *Ehrlichia* sp. clone from Tibet, the uncultured *Ehrlichia* sp. clone Dehong-18, and *Ehrlichia* sp. strain WHBMXZ-41, while all the strains from Liupanshui were 100% identical to these strains. Meanwhile, the *gltA* and *groEL* sequences were both closely related to *Ehrlichia* sp. strains detected in *R. microplus* from Wuhan city, Hubei Province (*Ehrlichia* sp. strain WHBMXZ-43, strain WHBMXZ-40, strain WHBMXZ-41, etc.), with similarities of 99.79–100%. In the phylogenetic trees, all the sequences from these strains were closely clustered (Figure 3).

### 2.4. Detection and Analysis of Anaplasma

Four *Anaplasma* species were identified in these samples: *A. marginale*, *A. ovis*, *A. capra*, and *Candidatus* Anaplasma boleense. *Anaplasma marginale* was detected in both Bijie (43.48%, 30/69) and Liupanshui (1.59%, 2/126) (Table 1). The representative strains Bijie15 and Liupanshui24 were both closely related to *A. marginale* strains, with the similarities of key genes varying from 99.78% to 100%. Alongside for *A. marginale*, *Candidatus* Anaplasma boleense was identified in ticks from Bijie (8.70%, 6/69). The BLASTn results showed that these strains were mostly related to strains reported in Wuhan City, Hubei Province. However, some variations were observed. Although the 16S genes had as high as 99.91–100% homology to *Candidatus* Anaplasma boleense strain WHBMXZ-151, strain WHANSA-29, and strain WHANSP-48, the recovered *gltA* sequences were 99.86% identical to strain WHBMXZ-139 but had similarities of lower than 97.75% to any other *Candidatus* Anaplasma boleense strains. As for the *groEL* genes, all the strains were only 99.10% identical to strain WHBMXZ-139, strain WHBMXZ-151, and strain WHBMXZ-45. In the phylogenetic tree based on *groEL*, the strains in this study formed a distinct cluster, indicating that this species has evolved for a long time in this area.

**Figure 4:**
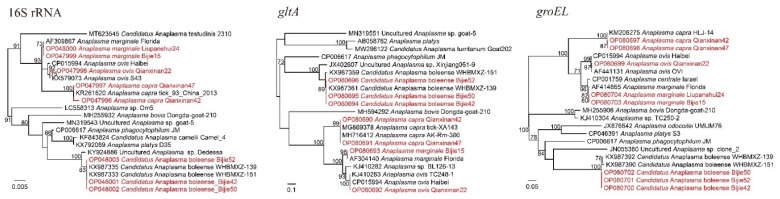
Phylogenetic trees constructed by PhyML 3.0 software (Sourced by Stéphane Guindon in Montpellier, France) (GTR model) based on the nucleotide sequences of 16S rRNA (1099–1299 bp) and the *gltA* (536–911 bp) and *groEL* (779–1338 bp) genes of *Anaplasma* strains. Red: Sequences obtained in this study.

In ticks from Qianxinan, *A. ovis* (9.88%, 8/81) and *A. capra* (4.94%, 4/81) were detected (Table 1), both of which are human pathogens. The 16S, *gltA*, and *groEL* sequences were all closely related to the *A. capra* strains reported elsewhere in China, with similarities of 99.69–99.92%, 99.88–100%, and 99.70% to other strains, respectively. All these strains clustered with other *A. capra* strains and formed a clade in the phylogenetic trees (Figure 4).

All the obtained sequences have been submitted to the GenBank Database (the accession numbers are shown in Supplementary Table S1).

## 3. Discussion

In China, *R. microplus* has been recorded in 188 counties [11]. In a previous study, we identified various *Rickettsia*, *Anaplasma*, and *Ehrlichia* species in *R. microplus* ticks from Hainan, Yunnan, Guangxi, Sichuan, and Hubei Provinces in China [10,16]. In this study, one *Rickettsia*, four *Anaplasma*, and three *Ehrlichia* species in total were detected and characterized in Guizhou Province.

All ticks from the three locations had high positive rates of *Candidatus* Rickettsia jingxinensis. *Candidatus* Rickettsia jingxinensis is a spotted fever group *Rickettsia* widely distributed in China and other countries. After its first report in Jilin Province, Northeast China [17], this *Rickettsia* species has been reported in Shaanxi, Sichuan, Guangxi, and Yunnan Provinces in China [10,12,18], as well as some neighboring countries, including Korea, Thailand, and India (GenBank Accession No. MN463681.1–MN463706.1) [19,20]. In previous studies, remarkably high positive rates of this *Rickettsia* (as high as 69.7% in *H. longicornis* from Shaanxi Province) in some areas have been observed [18]. In this study, the high positive rates in all three locations suggest the possibility that this *Rickettsia* may be a symbiont of this ixodid. Furthermore, it has been indicated that different endosymbiotic *Rickettsia* species may be unable to co-infect the same organ of the same tick, which is called “interference” [21]. In this study, only high positive rates of *Candidatus* Rickettsia jingxinensis were observed, while no other *Rickettsia* species were detected. It would be interesting to know whether this has resulted from the interference effect of *Candidatus* Rickettsia jingxinensis. Although this *Rickettsia* has only been detected in ticks up until now and there is no solid evidence proving its ability to infect animals or humans, it is phylogenetically close to some *Rickettsia* species pathogenic to humans such as *R. japonica* and *R. heilongjiangensis*. Furthermore, a *gltA* sequence (KU853023) obtained from a patient in China with 99.91% nucleotide identity to Ca. R. jingxinensis has been submitted to the GenBank database, suggesting its possible human pathogenicity. For the reasons above, more attention should be paid to its pathogenicity and further studies are needed.

*Ehrlichia canis* is a tick-borne pathogen mainly vectored and transmitted by *Rhipicephalus sanguineus*. It has also been reported in some other tick species such as *R. microplus*, *R. linnaei*, *R. bursa*, etc. [10,22,23]. As the causative agent of canine monocytic ehrlichiosis (CME), *E. canis* is one of the most prevalent tick-borne pathogens infecting dogs worldwide. Furthermore, it also infects other animals such as goats, sheep, deer, and red foxes [24,25,26]. As early as 1989, *E. canis* was reported to infect humans and cause a series of symptoms in the United States [27]. In recent years, more cases of infection have been reported in other countries such as Costa Rica [28]. In this study, *E. canis* was observed in ticks collected from goats in Qianxinan, suggesting that a tick–goat–tick cycle may exist in this area. Genetic and phylogenetic analysis indicated that these *E. canis* strains were closely related to those previously reported in Baise City in Guangxi Province [10], and they represented a variant that is different from most *E. canis* strains. 

*Ehrlichia minasensis*, an *Ehrlichia* species closely related to *E. canis*, has been reported in China, Canada, Brazil, Malaysia, Ethiopia, and South Africa, suggesting its worldwide distribution [29]. It has long been considered the etiologic agent of tick-borne bovine ehrlichiosis [29]. This is the first report that *E. minasensis* exists in Guizhou Province. Notably, the 16S sequences of all the *E. minasensis* strains in this study were more genetically related to those of other *Ehrlichia* species, indicating that some genetic recombination may have occurred.

In total, four *Anaplasma* species were detected in these samples, namely *A. capra*, *A. ovis*, *A. marginale*, and *Candidatus* Anaplasma boleense. In previous studies, *R. microplus* has been recognized as the vector of *A. marginale*, the agent of bovine anaplasmosis [30]. In this study, *A. marginale* had a high positive rate (30/69, 43.48%) in ticks collected from Bijie City, indicating that *A. marginale* is circulating in this area. Its potential effects on local husbandry should be considered. Of note, two zoonotic pathogens, *A. capra* and *A. ovis*, were detected, which occasionally infect humans and cause a series of symptoms [31,32]. In previous studies, the prevalence of *A. capra* and *A. ovis* was reported in other locations in Guizhou Province. Our results confirmed the existence of these pathogens and further proved their wide distribution in Guizhou Province, despite the relatively low positive rates and the low anthropophily of *R. microplus*.

In summary, this study contributed to our knowledge on the species’ abundance and the genetic diversity of Rickettsiales bacteria in Guizhou Province. Notably, some of these bacteria have been reported to infect humans. Considering the spatial proximity and close contact between humans and the ticks’ hosts, these results may indicate the potential risk of human exposure.

## 4. Methods

### 4.1. Sample Collection and DNA Extraction

From August to October 2021, 276 ticks were collected from 50 domestic animals (26 cattle and 24 goats) in Bijie City, Liupanshui City, and Qianxinan Bouyei-and-Miao Autonomous Prefecture (Supplementary Material Figure S2). In August 2021, 14 ticks from 3 cattle (*Bos taurus*) and 67 ticks from 24 goats (*Capra hircus*) were collected in Puan County of Qianxinan Bouyei-and-Miao Autonomous Prefecture (25.78° N 104.95° E). In September, 126 ticks were collected from 17 cattle in Liuzhi Special District of Liupanshui City (26.20° N 105.48° E,). In October 2021, 69 ticks were collected from six cattle in Qianxi County of Bijie City (27.01° N, 106.03° W). The ticks were carefully removed from the body surfaces of the animals using tweezers. All the ticks were morphologically identified and then individually screened for tick-borne pathogen species [33]. In addition, in order to confirm the identification of the tick species, a randomly chosen subsample was confirmed by amplifying and sequencing the *COI* gene [10]. In total, 26 ticks (26/276, 9.42%) were randomly selected from the three locations (Bijie: 3 ticks; Liupanshui: 10 ticks; Qianxinan: 13 ticks) for molecular confirmation. Before DNA extraction, each tick was washed three times using phosphate buffered saline (PBS) to remove environmental contamination and then thoroughly ground in a mortar with PBS (100 uL). The homogenates were manually placed in 1.5 mL Eppendorf tubes, and the DNA was extracted using Omega Mollusc DNA extraction kits (Omega Bio-Tek, Norcross, GA, USA). The DNA was eluted in 60 μL of an elution buffer and then kept in a freezer at −80 °C before molecular detection.

### 4.2. Molecular Detection of Rickettsiale

All the DNA samples were screened for the existence of Rickettsiales by amplifying conserved regions of the 16S rRNA gene by nested or semi-nested PCR. *Rickettsia* was detected using the protocol previously shown in [10], generating approximately 900 bp of PCR product. The Anaplasmataceae bacteria including *Ehrlichia*, and *Anaplasma* were detected using the primers as shown [15], generating approximately 400 bp of PCR product. Here, ddH_2_O was set as the negative control for each PCR. All the PCR products were electrophoresed in 1.0% agarose gels, and the PCR products that met the expected length were subjected to Sanger sequencing. The 16S sequences thus obtained were aligned with reference sequences in the GenBank database using BLASTn to initially determine the bacterial species or genus.

### 4.3. Amplification and Analysis of Key Genes

To exactly determine the bacterial species and further characterize the detected strains, representative strains were selected from the positive samples, and longer 16S fragments (1184 bp for *Rickettsia*, 1119–1250 bp for *Ehrlichia*, and 1099–1299 bp for *Anaplasma*) were amplified from these using primers [10,18]. The citrate synthase gene (*gltA*) and 60 kDa chaperonin (*groEL*) genes were also amplified [10,18,34] for all the representative *Rickettsia*, *Ehrlichia*, and *Anaplasma* strains. For the *Rickettsia* strains, the outer membrane protein A (*ompA*) sequences were additionally obtained using primers as shown in [18]. All the primers are shown in Supplementary Material Table S2.

### 4.4. Sequence and Phylogenetic Analysis

All the obtained sequences were assembled and edited using BioEdit software (North Carolina State University) and then aligned with sequences in the GenBank database using BLASTn to determine the nucleotides’ similarity. For phylogenetic analysis, all the recovered sequences were aligned with the reference sequences using the ClustalW method with the MEGA program, version 5.2 [35]. Representative strains were selected from most, if not all, validated *Rickettsia*, *Ehrlichia*, and *Anaplasma* species as reference sequences. Due to the limited quantity of validated *Ehrlichia* and *Anaplasma* species, some unvalidated species were also included. A substitution model test was performed to determine the phylogenetic model with the best fit. Maximum likelihood (ML) trees based on the aligned sequences were constructed by the GTR+I+G model using PhyML v3.2 [36]. All the trees were rooted at the mid-point.

## Figures and Tables

**Table 1:** Positive rates of Rickettsiales in 276 *Rhipicephalus microplus* ticks in three locations (Qianxinan, Liupanshui, and Bijie) of Guizhou Province, 2021.

Rickettsiales Species		Qianxinan	Liupanshui	Bijie	Total
*Rickettsia*	*Candidatus* Rickettsia jingxinensis	72/81 (88.89%)	126/126 (100.00%)	69/69 (100.00%)	267/276 (96.74%)
*Ehrlichia*	*Ehrlichia canis*	17/81 (20.99%)	0/126 (0.00%)	0/69 (0.00%)	16/276 (6.16%)
	*Ehrlichia minasensis*	2/81 (2.47%)	7/126 (5.56%)	0/69 (0.00%)	9/276 (3.26%)
	*Ehrlichia* sp.	0/81 (0.00%)	5/126 (3.97%)	7/69 (10.14%)	12/276 (4.35%)
*Anaplasma*	*Anaplasma capra*	4/81 (4.94%)	0/126 (0.00%)	0/69 (0.00%)	4/276 (1.45%)
	*Anaplasma marginale*	0/81 (0.00%)	2/126 (1.59%)	30/69 (43.48%)	32/276 (11.59%)
	*Anaplasma ovis*	8/81 (9.88%)	0/126 (0.00%)	0/69 (0.00%)	8/276 (2.90%)
	*Candidatus* Anaplasma boleense	0/81 (0.00%)	0/126 (0.00%)	6/69 (8.70%)	6/276 (2.17%)

## Data Availability

All the sequence files are available from the NCBI database (the accession numbers are shown in Table S1).

## Supplementary Materials

The following supporting information can be downloaded at: https://www.mdpi.com/article/10.3390/pathogens11101108/s1. Table S1. GenBank numbers of the *Rickettsia*, *Anaplasma*, and *Ehrlichia* sequences obtained in this study. Table S2. The primers used for amplification of the 16S, *gltA*, *groEL*, and *ompA* genes from *Rickettsia*, *Anaplasma*, and *Ehrlichia* by nested PCR or hemi-nested PCR. Figure S1. Phylogenetic trees constructed by PhyML 3.0 software (GTR model) based on concatenated sequences of the *Rickettsia*, *Ehrlichia*, and *Anaplasma* strains. Figure S2. Map showing the locations where the samples were collected: Puan County of Qianxinan Bouyei-and-Miao Autonomous Prefecture; Liuzhi Special District of Liupanshui City; and Qianxi County of Bijie City.

## References

[1] Mansfield K.L., Jizhou L., Phipps L.P., Johnson N. (2017). Emerging ttick-borne viruses in the twenty-first century. Front. Cell. Infect. Microbiol..

[2] Madison-Antenucci S., Kramer L.D., Gebhardt L.L., Kauffman E. (2020). Emerging tick-borne diseases. Clin. Microbiol. Rev..

[3] Mediannikov O., Fenollar F. (2014). Looking in ticks for human bacterial pathogens. Microb. Pathog..

[4] Rafinejad J., Choubdar N., Oshaghi M., Piazak N., Satvat T., Mohtarami F., Barmaki A. (2011). Detection of *Borrelia persica* Infection in *Ornithodoros tholozani* using PCR targeting *rrs* gene and xenodiagnosis. Iran. J. Public Health.

[5] de la Fuente J., Estrada-Pena A., Venzal J.M., Kocan K.M., Sonenshine D.E. (2008). Overview: Ticks as vectors of pathogens that cause disease in humans and animals. Front. Biosci..

[6] Kamani J., Apanaskevich D.A., Gutiérrez R., Nachum-Biala Y., Baneth G., Harrus S. (2017). Morphological and molecular identification of *Rhipicephalus* (*Boophilus*) *microplus* in Nigeria, West Africa: A threat to livestock health. Exp. Appl. Acarol..

[7] Ali A., Khan M.A., Zahid H., Yaseen P.M., Qayash Khan M., Nawab J., Ur Rehman Z., Ateeq M., Khan S., Ibrahim M. (2019). Seasonal dynamics, record of ticks infesting humans, wild and domestic animals and molecular phylogeny of *Rhipicephalus microplus* in Khyber Pakhtunkhwa Pakistan. Front. Physiol..

[8] Cordeiro M.D., de Azevedo Baêta B., Cepeda P.B., Teixeira R.C., Ribeiro C.C.D.U., de Almeida Valim J.R., Pinter A., da Fonseca A.H. (2018). Experimental infection of *Rickettsia parkeri* in the *Rhipicephalus microplus* tick. Ticks Tick-Borne Dis..

[9] Zhang X.L., Deng Y.P., Yang T., Li L.Y., Cheng T.Y., Liu G.H., Duan D.Y. (2022). Metagenomics of the midgut microbiome of *Rhipicephalus microplus* from China. Parasites Vectors.

[10] Lu M., Tian J., Pan X., Qin X., Wang W., Chen J., Guo W., Li K. (2022). Identification of *Rickettsia* spp., *Anaplasma* spp., and an *Ehrlichia canis*-like agent in *Rhipicephalus microplus* from Southwest and South-Central China. Ticks Tick-Borne Dis..

[11] Zhao G.P., Wang Y.X., Fan Z.W., Ji Y., Liu M.J., Zhang W.H., Li X.L., Zhou S.X., Li H., Liang S. (2021). Mapping ticks and tick-borne pathogens in China. Nat. Commun..

[12] Peng Y., Wang K., Zhao S., Yan Y., Wang H., Jing J., Jian F., Wang R., Zhang L., Ning C. (2018). Detection and phylogenetic characterization of *Anaplasma capra*: An emerging pathogen in sheep and goats in China. Front. Cell. Infect. Microbiol..

[13] Wang Q., Guo W.B., Pan Y.S., Jiang B.G., Du C.H., Que T.C., Zhan L., Wu J.H., Yu M.H., Cui X.M. (2021). Detection of novel spotted fever group Rickettsiae (Rickettsiales: Rickettsiaceae) in ticks (Acari: Ixodidae) in Southwestern China. J. Med. Entomol..

[14] Liu Z., Ma M., Wang Z., Wang J., Peng Y., Li Y., Guan G., Luo J., Yin H. (2012). Molecular survey and genetic identification of *Anaplasma* species in goats from central and southern China. Appl. Environ. Microbiol..

[15] Lu M., Tian J., Zhao H., Jiang H., Qin X., Wang W., Li K. (2022). Molecular Survey of Vector-Borne Pathogens in Ticks, Sheep Keds, and Domestic Animals from Ngawa, Southwest China. Pathogens.

[16] Lu M., Tang G.P., Bai X.S., Qin X.C., Wang W., Guo W.P., Li K. (2021). Molecular Detection of tick-borne pathogens in ticks collected from Hainan Island, China. Biomed. Environ. Sci..

[17] Liu H., Li Q., Zhang X., Li Z., Wang Z., Song M., Wei F., Wang S., Liu Q. (2016). Characterization of rickettsiae in ticks in northeastern China. Parasites Vectors.

[18] Guo W.P., Wang Y.H., Lu Q., Xu G., Luo Y., Ni X., Zhou E.M. (2019). Molecular detection of spotted fever group rickettsiae in hard ticks, northern China. Transbound. Emerg. Dis..

[19] Park H.J., Kim J., Choi Y.J., Kim H.C., Klein T.A., Chong S.T., Jiang J., Richards A.L., Jang W.J. (2021). Tick-borne rickettsiae in Midwestern region of Republic of Korea. Acta Trop..

[20] Takhampunya R., Korkusol A., Pongpichit C., Yodin K., Rungrojn A., Chanarat N., Promsathaporn S., Monkanna T., Thaloengsok S., Tippayachai B. (2019). Metagenomic Approach to Characterizing Disease Epidemiology in a Disease-Endemic Environment in Northern Thailand. Front. Microbiol..

[21] Gurfield N., Grewal S., Cua L.S., Torres P.J., Kelley S.T. (2017). Endosymbiont interference and microbial diversity of the Pacific coast tick, *Dermacentor occidentalis*, in San Diego County, California. PeerJ.

[22] Kaewmongkol G., Lukkana N., Yangtara S., Kaewmongkol S., Thengchaisri N., Sirinarumitr T., Jittapalapong S., Fenwick S.G. (2017). Association of *Ehrlichia canis*, hemotropic *Mycoplasma* spp. and *Anaplasma platys* and severe anemia in dogs in Thailand. Vet. Microbiol..

[23] Neave M.J., Mileto P., Joseph A., Reid T.J., Scott A., Williams D.T., Keyburn A.L. (2022). Comparative genomic analysis of the first *Ehrlichia canis* detections in Australia. Ticks Tick-Borne Dis..

[24] Chisu V., Loi F., Mura L., Tanda A., Chessa G., Masala G. (2021). Molecular detection of *Theileria sergentii*/*orientalis*/*buffeli* and *Ehrlichia canis* from aborted ovine and caprine products in Sardinia, Italy. Vet. Med. Sci..

[25] Bezerra-Santos M.A., Nguyen V.L., Iatta R., Manoj R.R.S., Latrofa M.S., Hodžić A., Dantas-Torres F., Mendoza-Roldan J.A., Otranto D. (2021). Genetic variability of *Ehrlichia canis* TRP36 in ticks, dogs, and red foxes from Eurasia. Vet. Microbiol..

[26] Li Y., Chen Z., Liu Z., Liu J., Yang J., Li Q., Li Y., Luo J., Yin H. (2016). Molecular survey of *Anaplasma* and *Ehrlichia* of red deer and Sika deer in Gansu, China in 2013. Transbound. Emerg. Dis..

[27] Conrad M.E. (1989). *Ehrlichia canis*: A tick-borne rickettsial-like infection in humans living in the southeastern United States. Am. J. Med. Sci..

[28] Bouza-Mora L., Dolz G., Solórzano-Morales A., Romero-Zuñiga J.J., Salazar-Sánchez L., Labruna M.B., Aguiar D.M. (2017). Novel genotype of *Ehrlichia canis* detected in samples of human blood bank donors in Costa Rica. Ticks Tick-Borne Dis..

[29] Moura de Aguiar D., Pessoa Araújo Junior J., Nakazato L., Bard E., Aguilar-Bultet L., Vorimore F., Leonidovich Popov V., Moleta Colodel E., Cabezas-Cruz A. (2019). Isolation and characterization of a novel pathogenic strain of *Ehrlichia minasensis*. Microorganisms.

[30] Ribeiro M.F., Lima J.D. (1996). Morphology and development of *Anaplasma marginale* in midgut of engorged female ticks of *Boophilus microplus*. Vet. Parasitol..

[31] Li H., Zheng Y.C., Ma L., Jia N., Jiang B.G., Jiang R.R., Huo Q.B., Wang Y.W., Liu H.B., Chu Y.L. (2015). Human infection with a novel tick-borne *Anaplasma* species in China: A surveillance study. Lancet Infect. Dis..

[32] Chochlakis D., Ioannou I., Tselentis Y., Psaroulaki A. (2010). Human anaplasmosis and *Anaplasma ovis* variant. Emerg. Infect. Dis..

[33] Namgyal J., Lysyk T.J., Couloigner I., Checkley S., Gurung R.B., Tenzin T., Dorjee S., Cork S.C. (2021). Identification, Distribution, and Habitat Suitability Models of Ixodid Tick Species in Cattle in Eastern Bhutan. Trop. Med. Infect. Dis..

[34] Guo W.P., Tian J.H., Lin X.D., Ni X.B., Chen X.P., Liao Y., Yang S.Y., Dumler J.S., Holmes E.C., Zhang Y.Z. (2016). Extensive genetic diversity of Rickettsiales bacteria in multiple mosquito species. Sci. Rep..

[35] Kumar S., Stecher G., Tamura K. (2016). MEGA7: Molecular Evolutionary Genetics Analysis Version 7.0 for Bigger Datasets. Mol. Biol. Evol..

[36] Guindon S., Delsuc F., Dufayard J.F., Gascuel O. (2009). Estimating maximum likelihood phylogenies with PhyML. Methods Mol. Biol..

