# Supplementary material for: Diversity of Rickettsiales in Rhipicephalus microplus Ticks Collected in Domestic Ruminants in Guizhou Province, China
Source: Pathogens. 2022 Sep 27;11(10):1108. doi: 10.3390/pathogens11101108 (PMC9607482; doi:10.3390/pathogens11101108)
Supplement: Supplementary file 1 [file pathogens-11-01108-s001.zip › Figures S1 and S2.pdf]

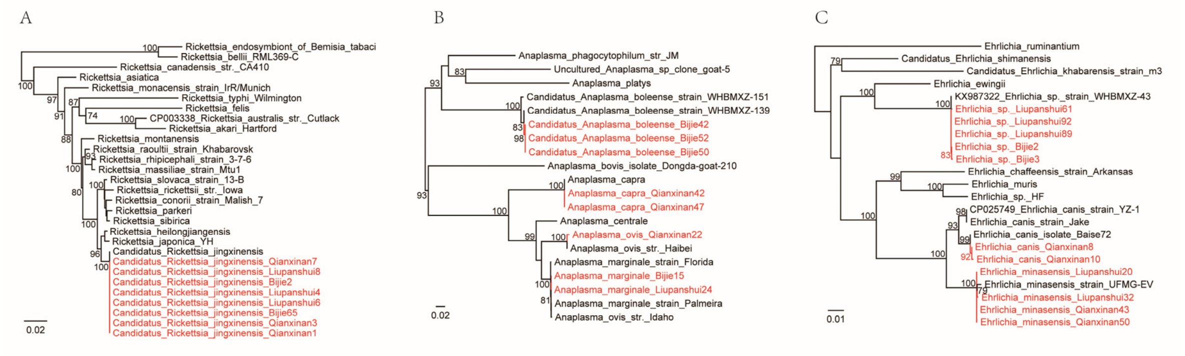

Figure S1. Phylogenetic trees constructed by PhyML 3.0 software (GTR model) based on concatenated sequences of the *Rickettsia*, *Ehrlichia*, and *Anaplasma* strains

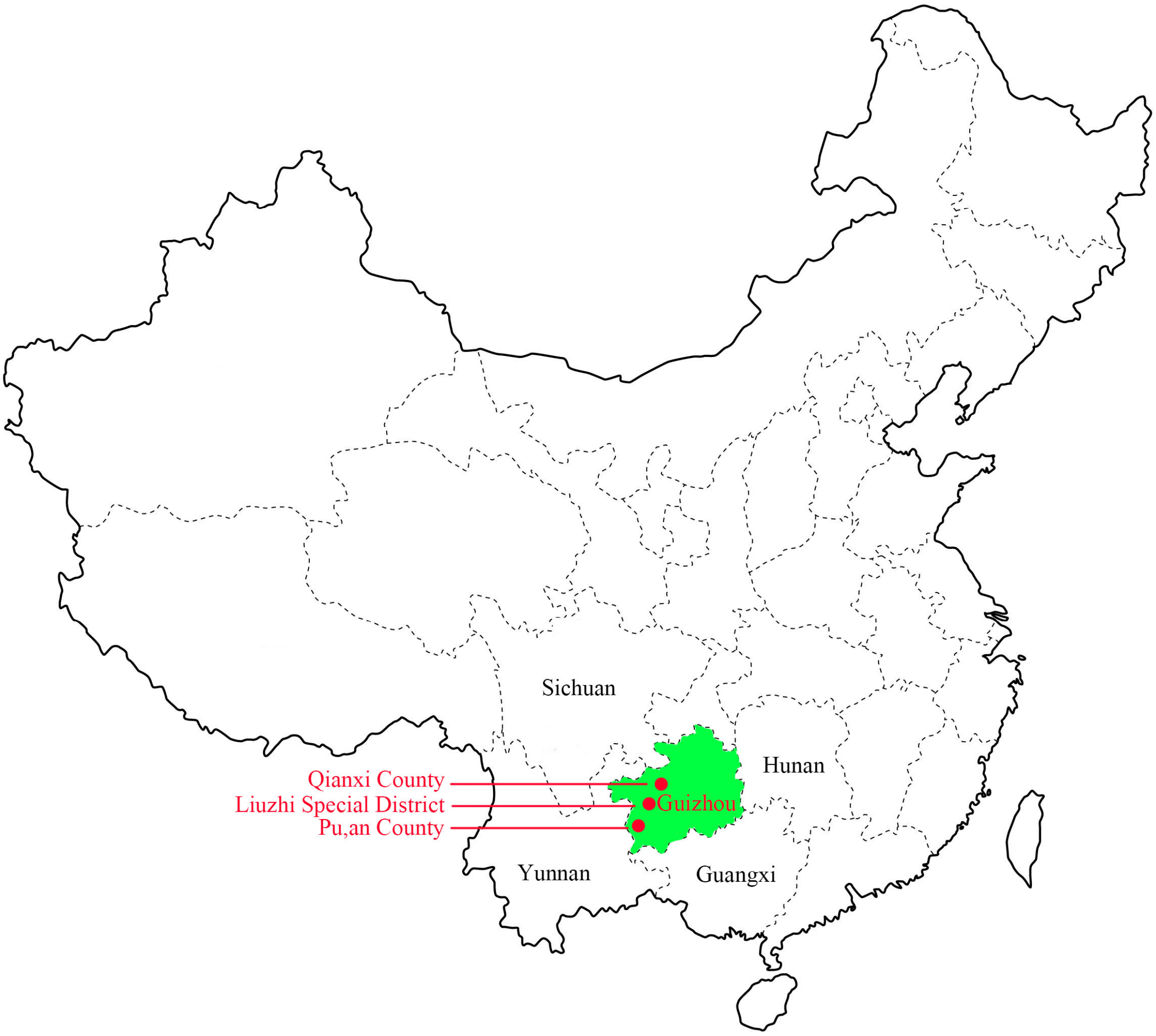

Figure S2. Map showing the locations where the samples were collected: Puan County of Qianxinan Bouyei-and-Miao Autonomous Prefecture; Liuzhi Special District of Liupanshui City; and Qianxi County of Bijie City
