# Supplementary material for: Diversity of Rickettsiales in Rhipicephalus microplus Ticks Collected in Domestic Ruminants in Guizhou Province, China
Source: Pathogens. 2022 Sep 27;11(10):1108. doi: 10.3390/pathogens11101108 (PMC9607482; doi:10.3390/pathogens11101108)
Supplement: Supplementary file 1 [file pathogens-11-01108-s001.zip › Table S1.pdf]

Table S1. Genbank numbers of *Rickettsia*, *Anaplasma*, and *Ehrlichia* sequences obtained in this study.

	Gene	Genbank numbers	Bacterial strain
1	16S	OP047977	Candidatus_Rickettsia_jingxinensis_Qianxinan1
2	16S	OP047978	Candidatus_Rickettsia_jingxinensis_Qianxinan3
3	16S	OP047979	Candidatus_Rickettsia_jingxinensis_Qianxinan7
4	16S	OP047980	Candidatus_Rickettsia_jingxinensis_Bijie2
5	16S	OP047981	Candidatus_Rickettsia_jingxinensis_Bijie65
6	16S	OP047982	Candidatus_Rickettsia_jingxinensis_Liupanshui4
7	16S	OP047983	Candidatus_Rickettsia_jingxinensis_Liupanshui6
8	16S	OP047984	Candidatus_Rickettsia_jingxinensis_Liupanshui8
9	16S	OP047985	Ehrlichia_canis_Qianxinan8
10	16S	OP047986	Ehrlichia_canis_Qianxinan10
11	16S	OP047987	Ehrlichia_minasensis_Qianxinan43
12	16S	OP047988	Ehrlichia_minasensis_Qianxinan50
13	16S	OP047989	Ehrlichia_sp._Bijie2
14	16S	OP047990	Ehrlichia_sp._Bijie3
15	16S	OP047991	Ehrlichia_minasensis_Liuzhi20
16	16S	OP047992	Ehrlichia_minasensis_Liuzhi32
17	16S	OP047993	Ehrlichia_sp._Liuzhi61
18	16S	OP047994	Ehrlichia_sp._Liuzhi89
19	16S	OP047995	Ehrlichia_sp._Liuzhi92
20	16S	OP047996	Anaplasma_capra_Qianxinan42
21	16S	OP047997	Anaplasma_capra_Qianxinan47
22	16S	OP047998	Anaplasma_ovis_Qianxinan22
23	16S	OP047999	Anaplasma_marginale_Bijie15
24	16S	OP048000	Anaplasma_marginale_Liuzhi24
25	16S	OP048001	Candidatus_Anaplasma_boleense_Bijie42
26	16S	OP048002	Candidatus_Anaplasma_boleense_Bijie50
27	16S	OP048003	Candidatus_Anaplasma_boleense_Bijie52
28	gltA	OP080652	Candidatus_Rickettsia_jingxinensis_Qianxinan1
29	gltA	OP080653	Candidatus_Rickettsia_jingxinensis_Qianxinan3
30	gltA	OP080654	Candidatus_Rickettsia_jingxinensis_Qianxinan7
31	gltA	OP080655	Candidatus_Rickettsia_jingxinensis_Bijie2
32	gltA	OP080656	Candidatus_Rickettsia_jingxinensis_Bijie65
33	gltA	OP080657	Candidatus_Rickettsia_jingxinensis_Liupanshui4
34	gltA	OP080658	Candidatus_Rickettsia_jingxinensis_Liupanshui6
35	gltA	OP080659	Candidatus_Rickettsia_jingxinensis_Liupanshui8
36	gltA	OP080668	Ehrlichia_canis_Qianxinan8
37	gltA	OP080669	Ehrlichia_canis_Qianxinan10
38	gltA	OP080670	Ehrlichia_minasensis_Qianxinan43

39	gltA	OP080671	Ehrlichia_minasensis_Qianxinan50
40	gltA	OP080672	Ehrlichia_sp._Bijie2
41	gltA	OP080673	Ehrlichia_sp._Bijie3
42	gltA	OP080674	Ehrlichia_minasensis_Liuzhi20
43	gltA	OP080675	Ehrlichia_minasensis_Liuzhi32
44	gltA	OP080676	Ehrlichia_sp._Liuzhi61
45	gltA	OP080677	Ehrlichia_sp._Liuzhi89
46	gltA	OP080678	Ehrlichia_sp._Liuzhi92
47	gltA	OP080690	Anaplasma_capra_Qianxinan42
48	gltA	OP080691	Anaplasma_capra_Qianxinan47
49	gltA	OP080692	Anaplasma_ovis_Qianxinan22
50	gltA	OP080693	Anaplasma_marginale_Bijie15
51	gltA	OP080694	Candidatus_Anaplasma_boleense_Bijie42
52	gltA	OP080695	Candidatus_Anaplasma_boleense_Bijie50
53	gltA	OP080696	Candidatus_Anaplasma_boleense_Bijie52
54	groEL	OP080660	Candidatus_Rickettsia_jingxinensis_Qianxinan1
55	groEL	OP080661	Candidatus_Rickettsia_jingxinensis_Qianxinan3
56	groEL	OP080662	Candidatus_Rickettsia_jingxinensis_Qianxinan7
57	groEL	OP080663	Candidatus_Rickettsia_jingxinensis_Bijie2
58	groEL	OP080664	Candidatus_Rickettsia_jingxinensis_Bijie65
59	groEL	OP080665	Candidatus_Rickettsia_jingxinensis_Liupanshui4
60	groEL	OP080666	Candidatus_Rickettsia_jingxinensis_Liupanshui6
61	groEL	OP080667	Candidatus_Rickettsia_jingxinensis_Liupanshui8
62	groEL	OP080679	Ehrlichia_canis_Qianxinan8
63	groEL	OP080680	Ehrlichia_canis_Qianxinan10
64	groEL	OP080681	Ehrlichia_minasensis_Qianxinan43
65	groEL	OP080682	Ehrlichia_minasensis_Qianxinan50
66	groEL	OP080683	Ehrlichia_sp._Bijie2
67	groEL	OP080684	Ehrlichia_sp._Bijie3
68	groEL	OP080685	Ehrlichia_minasensis_Liuzhi20
69	groEL	OP080686	Ehrlichia_minasensis_Liuzhi32
70	groEL	OP080687	Ehrlichia_sp._Liuzhi61
71	groEL	OP080688	Ehrlichia_sp._Liuzhi89
72	groEL	OP080689	Ehrlichia_sp._Liuzhi92
73	groEL	OP080697	Anaplasma_capra_Qianxinan42
74	groEL	OP080698	Anaplasma_capra_Qianxinan47
75	groEL	OP080699	Anaplasma_ovis_Qianxinan22
76	groEL	OP080703	Anaplasma_marginale_Bijie15
77	groEL	OP080704	Anaplasma_marginale_Liuzhi24
78	groEL	OP080700	Candidatus_Anaplasma_boleense_Bijie42
79	groEL	OP080701	Candidatus_Anaplasma_boleense_Bijie50
80	groEL	OP080702	Candidatus_Anaplasma_boleense_Bijie52
