# Supplementary material for: Diversity of Rickettsiales in Rhipicephalus microplus Ticks Collected in Domestic Ruminants in Guizhou Province, China
Source: Pathogens. 2022 Sep 27;11(10):1108. doi: 10.3390/pathogens11101108 (PMC9607482; doi:10.3390/pathogens11101108)
Supplement: Supplementary file 1 [file pathogens-11-01108-s001.zip › Table S2.pdf]

Table S2 The primers used for amplification of 16S, *gltA*, *groEL*, and *ompA* genes from *Rickettsia*, *Anaplasma*, and *Ehrlichia* by nested PCR or hemi-nested PCR.

Primer	Cycle	Bacteria	Gene	Sequence	Anticipated amplicon length	Reference
Eh1	1	Anaplasmataceae	16S	5-AACGAACGCTGGCGGCAAGC-3	450 bp	Guo et al., 2019
Eh2	1	Anaplasmataceae	16S	5-AGTAYCGRACCAGATAGCCGC-3		
Eh3	2	Anaplasmataceae	16S	5-TGCATAGGAATCTACCTAGTAG-3		
Eh4	2	Anaplasmataceae	16S	5-CTAGGAATTCCGCTATCCTCT-3		
Ric-F	1, 2	*Rickettsia*	16S	5-YTACGGAATAACTTTTAGAAA-3	900 bp	Lu et al., 2022
Ric-R1	1	*Rickettsia*	16S	5-CATGATGACTTGACRTCGT-3		
Ric-R2	2	*Rickettsia*	16S	5-CATCTCACGACACGAGCTG-3		
fD1	1, 2	*Rickettsia,* Anaplasmataceae	16S	5-AGAGTTTGATCCTGGCTCAG-3	1100-1300 bp	Guo et al., 2019
rp2	1	*Rickettsia,* Anaplasmataceae	16S	5-ACGGCTACCTTGTTACGACTT-3		
Eh2	2	*Rickettsia,* Anaplasmataceae	16S	5-AGTAYCGRACCAGATAGCCGC-3		
Ric-glt-F1	1	*Rickettsia*	*gltA*	5-CCGGGYTTTATGTCTACTGC-3	1100 bp	Guo et al., 2019
Ric-glt-F2	2	*Rickettsia*	*gltA*	5-CTTTATGTCTACTGCKTCTTG-3		

Ric-glt-R	1, 2	*Rickettsia*	*gltA*	5-AGCTGTCTWGGTCTGCTGATT-3		
Ric-gro-F1	1	*Rickettsia*	*groEL*	5-CCATTACATGATAGAATTGCAAT-3	1100 bp	Guo et al., 2019
Ric-gro-F2	2	*Rickettsia*	*groEL*	5-GAATTGCAATAAAGCCTATCG-3		
Ric-gro-R	1, 2	*Rickettsia*	*groEL*	5-CCATCATTGCTTTTCTTCTATC-3		
Bole-gltA-F1	1	*Ca. A. boleense*	*gltA*	5-GYAGCATAGCGYATTTGTTGTTG-3	700 bp	This study
Bole-gltA-F2	2	*Ca. A. boleense*	*gltA*	5-TTGAGAGATGAGTATGTYCTACC-3		
Bole-gltA-R	1, 2	*Ca. A. boleense*	*gltA*	5-TCAACRTTAGGGTAAAGCTTGCG-3		
Capra-glt-F1	1	*Anaplasma capra*	*gltA*	5-ATGATCCGGGGTTCCTGTC-3	800 bp	Guo et al., 2019
Capra-glt-F2	2	*Anaplasma capra*	*gltA*	5-TGCAGGTCTGAGATAACCT-3		
Capra-glt-R	1, 2	*Anaplasma capra*	*gltA*	5-TACAATACCGGAGTAAAAGT-3		
Ovis-F	1, 2	*Anaplasma ovis*	*gltA*	5-GTGAGCTTGCCGACTTTGT-3	560 bp	Guo et al., 2019
Ovis-R1	1	*Anaplasma ovis*	*gltA*	5-GTTCTTGTAGACYCTGTGG-3		
Ovis-R2	2	*Anaplasma ovis*	*gltA*	5-ATGAGTCTCACTCCGCTCT-3		
Ana-glt-F1	1	*Anaplasma marginale*	*gltA*	5-CATCCNATGGCTATTYTCAT-3	900 bp	Lu et al., 2022
Ana-glt-R1	1	*Anaplasma marginale*	*gltA*	5-ACTATACCKGAGTAAAAGTC-3		
Ana-glt-F2	2	*Anaplasma marginale*	*gltA*	5-GAYCACGARCARAATGCTTC-3		
Ana-glt-R2	2	*Anaplasma marginale*	*gltA*	5-GAGTAAAAGTCGACRTTKGG-3		

Ana-gro-F1	1	*Anaplasma* sp.	*groEL*	5-GYCAGTGGGCTGGTAATGAA-3	1100 bp	Lu et al., 2022
Ana-gro-R1	1	*Anaplasma* sp.	*groEL*	5-CCWCCTGGTACWACACCTTC-3		
Ana-gro-F2	2	*Anaplasma* sp.	*groEL*	5-ATAGTYATGAAGGAGAGTGAT-3		
Ana-gro-R2	2	*Anaplasma* sp.	*groEL*	5-TCAACAGCAGCTCTAGTWG-3		
Bole-F1	1	*Ca. A. boleense*	*groEL*	5-CCGGAAATCACAAAAGACG-3	800 bp	This study
Bole-F2	2	*Ca. A. boleense*	*groEL*	5-GCTATAAAGTGATGAAGAGTATT-3		
Bole-R	1, 2	*Ca. A. boleense*	*groEL*	5-AATACTTTCGGAATTACTATCTAC-3		
Ehr-gltA-F1	1	*Ehrlichia*	*gltA*	5-TATGRTCRAAGAAGCAGTATT-3	1000 bp	Lu et al., 2022
Ehr-gltA-F2	2	*Ehrlichia*	*gltA*	5-GGAATATTAACTTATGATCC-3		
Ehr-gltA-R	1, 2	*Ehrlichia*	*gltA*	5-CTGACGTGGACGACATATCT-3		
Ehr-gro-F1	1	*Ehrlichia*	*groEL*	5-TGGGCTGGYAATGAAATTGA-3	1100 bp	Lu et al., 2022
Ehr-gro-F2	2	*Ehrlichia*	*groEL*	5-AACATGGCAAATGTAGTTGT-3		
Ehr-gro-R	1, 2	*Ehrlichia*	*groEL*	5-TCAACAGCAGCTCTAGTTG-3		
Rr190.70	1, 2	*Rickettsia*	*ompA*	5-ATGGCGAATATTTCTCCAAA-3	700 bp	Guo et al., 2019
Ric-R1	1	*Rickettsia*	*ompA*	5-ACCTACATTATCAAHGCCTGT-3		
Ric-R2	2	*Rickettsia*	*ompA*	5-ACCTSTTAATACTGCATTTRCAT-3		
